# Assessment of the performances of blood tests for the antemortem diagnosis of aspergillosis in wild or captive aquatic birds

**DOI:** 10.1186/s13567-026-01711-3

**Published:** 2026-02-03

**Authors:** Sabrina Vieu, Nicolas Soetart, Emilie Jeannès, Anne Lehebel, Nadine Brisseau, Pierre Cordier, Sylvie Laidebeure, Benjamin Lamglait, Julie Botman, Philippe Gourlay, Guillaume Desoubeaux, François Beaudeau, Aurélien Madouasse, Jacques Guillot

**Affiliations:** 1 Oniris, CHUV, 44300 Nantes, France; 2 Oniris, INRAE, BIOEPAR, 44300 Nantes, France; 3 IRF, IRCAT, Université d’Angers, 49000 Angers, France; 4 Oniris, LabOniris, 44300 Nantes, France; 5 Parc zoologique de Paris-Muséum national d’Histoire naturelle, 75012 Paris, France; 6 ZooParc de Beauval and Beauval Nature, 41110 St Aignan, France; 7 CHRU Tours, Parasitologie-Mycologie Médicale-Médecine Tropicale, 37000 Tours, France

**Keywords:** Birds, aspergillosis, electrophoresis, 3-hydroxybutyrate, beta-glucan, galactomannan, mannoprotein

## Abstract

The antemortem diagnosis of aspergillosis in birds remains a complex challenge. A variety of diagnostic methods are currently available, including direct detection of *Aspergillus* components, antibody-based assays, and nonspecific markers such as serum protein electrophoresis (SPE), but their diagnostic performances remain limited. The objective of this study was to assess and compare the performances of several diagnostic approaches, specifically, galactomannan index measurement, beta-D-glucan assay, 3-hydroxybutyrate quantification, SPE, and mannoprotein detection tests. A secondary objective was to develop a predictive model of aspergillosis incorporating optimal test thresholds identified in the first phase, combined with clinical signs. A total of 101 serum and 31 plasma samples were collected from 118 aquatic bird of various species in France. Birds were classified into three categories: control individuals (*n* = 88), suspected aspergillosis cases (*n* = 7), and confirmed cases (*n* = 23). While individually considered tests displayed limitations in specificity, predictive modeling revealed that elevated levels of 3-hydroxybutyrate above 0.52 mmol/L (Se = 96%, Sp = 51%) combined with beta-globulins above 6.90 g/L (Se = 78%, Sp = 51%), in conjunction with concurrent respiratory clinical signs, were significantly associated with the occurrence of aspergillosis. A multivariable logistic model combining these variables achieved excellent diagnostic performance, with AUCs up to 0.98 and sensitivity above 98%. These findings suggest that these parameters, particularly when considered alongside clinical signs, may serve as more reliable indicators for detecting aspergillosis in birds.

**Supplementary Information:**

The online version contains supplementary material available at 10.1186/s13567-026-01711-3.

## Introduction

*Aspergillus* species are saprophytic fungi that thrive in organic matter and spread through airborne spores. Birds can inhale *Aspergillus* spores, particularly in crowded or poorly ventilated conditions. Once inhaled, spores may germinate and initiate an infection process that can take advantage of immunosuppression or stress in the host [1]. *Aspergillus fumigatus* is the most pathogenic species. It has been reported in a wide range of host species, from backyard poultry to wild raptors [2, 3]. In wild bird populations, aspergillosis can contribute to population declines, particularly among species that are already facing threats from habitat loss, climate change, and other infectious diseases [3–5]. The clinical signs associated with avian aspergillosis can be variable, often including respiratory signs or general signs symptoms such as lethargy and decreased appetite [6]. Such a broad spectrum of clinical signs frequently overlaps with other avian diseases, complicating diagnosis and leading to potential misdiagnosis or delayed treatment. Furthermore, early stage infections may be asymptomatic or exhibit mild clinical signs, which can impair diagnosis [7]. Antemortem diagnosis is valuable, enabling timely treatment. Imaging techniques such as radiography and computed tomography can reveal respiratory anomalies indicative of infection, though they lack specificity and require supplementary tests such as endoscopy for accurate diagnosis [8, 9].

Blood sampling offers a possibility to detect biomarkers, though advancements in assay sensitivity and specificity remain necessary to reliably differentiate aspergillosis from other conditions. Blood markers for diagnosing avian aspergillosis are commonly derived from human medicine. With respect to this point, specific tests such as galactomannan (GM) and (1 → 3)-β-D-glucan (BDG), both components of the fungal cell wall, can indicate active infection. Protein electrophoresis can reveal changes in globulins associated with chronic infection [10, 11]. Detection of mannoprotein with an *Aspergillus* lateral-flow device (AspLFD) shows promising specificity and sensitivity, but it has only been evaluated in a small sample of birds of the same order [12]. Antibody detection relies on immune response and may signal *Aspergillus* either simple exposure or actual infection. High antibody titers may be found in healthy animal, implying that its reliability remains limited [13]. Detection of *Aspergillus* DNA by polymerase chain reaction (PCR) provides a specific and reliable method but must be performed directly from the lesion [14]. Together, these markers provide a multifaceted approach for aspergillosis diagnosis. However, these tests have rarely been applied and compared within the same cohort of birds, limiting the understanding of their relative efficacy and diagnostic potential.

In that context, this study aimed to provide key insights into the diagnostic performance and clinical applicability of various tools for avian aspergillosis. More specifically, our objectives were (i) to evaluate the accuracy of various biological tests (GM detection, BDG concentration, SPE, mannoprotein detection, and 3-hydroxybutyrate (3HB) measurement) for detecting avian aspergillosis and (ii) to develop a statistical model to support aspergillosis diagnosis to determine whether selected subsets of clinical signs and laboratory tests could be used as predictors of the disease state.

## Materials and methods

### Collection of biological samples and bird categorization

From September 2023 to December 2024, a veterinary network was engaged to collect serum and plasma samples from aquatic birds in France. Veterinary clinics, wildlife rehabilitation centers, and zoological parks were solicited. Biological samples were accompanied by detailed clinical records for each animal by the different structure. The birds were categorized into three groups: group A included clinically healthy individuals and birds with proven trauma but without evidence of aspergillosis (considered as controls). Birds in group A that presented with trauma showed no other clinical signs consistent with aspergillosis, nor radiographic evidence suggestive of this disease. This group also included animals that were euthanized and underwent post-mortem examination, with no macroscopic lesions compatible with aspergillosis. Group B included suspected aspergillosis cases, and group C confirmed aspergillosis cases. Suspected cases were categorized upon observation of general and respiratory signs and diagnostic imaging examination in favor of aspergillosis. Confirmed aspergillosis cases were based on histological and/or mycological evidence of *Aspergillus* infection. Mycological analysis was performed using Sabouraud dextrose agar, with incubation at 37 °C for 5 days. Fungal colonies were then examined microscopically using lactophenol cotton blue stain to assess the morphology of conidiophores and conidia for identification. Histological confirmation was based on the observation of fungal hyphae in tissue sections sampled from the lungs or air sacs. These tissues were stained using hematoxylin and eosin (H&E), Grocott-Gomori’s methenamine silver (GMS), or periodic acid–Schiff (PAS) stains to enhance fungal structure visualization.

Respiratory signs included: bilateral conjunctivitis, nasal discharge, sneezing, rhinitis, sinusitis, respiratory crackles, increased respiratory sounds, open-beak breathing, voice loss/alteration, and/or signs of general health, as well as lethargy, anorexia/dysorexia, regurgitation, crop stasis, weight loss, cyanotic, mucous membranes, pale mucous membranes, polyuria-polydipsia, and polypnea. Diagnostic imaging consisted of radiographs with modification of air sacs (thickening, focal density, or asymmetry) and lungs (focal density or pulmonary consolidation) and computed tomography scan with modification of trachea (thickening, luminal fluid accumulation, wall mineralization), bronchi and syrinx (occlusion, distortion, and wall thickening), lungs (parenchymal pulmonary density abnormalities, focal consolidation), and air sacs (wall thickening, presence of luminal fluid or soft tissue, focal consolidation).

All plasma and serum samples were initially collected at different institutions and stored at −20 °C for up to 3 months before analysis. Samples showing significant deterioration (e.g., hemolysis or lipemia) were excluded from the study. Veterinarians collected blood samples prior to euthanasia, which allowed for subsequent confirmation or exclusion of aspergillosis through necropsy, histopathology, and mycology. Birds without aspergillosis lesions at necropsy were classified as controls, as were birds sampled during routine health checks or vaccination procedures in the absence of clinical signs. To maximize the sample size, older retrospective samples were also required to the various institutions, including some banked for several years prior to the study period. In some retrospective cases, histological and mycological cultures were not always available for these cases.

### Blood tests

After thawing, hydroxybutyrate levels and protein concentration were measured using an automated clinical chemistry analyzer (RX Daytona, Randox Laboratories Ltd.) with reagents provided by the manufacturer. Calibration procedures followed the manufacturer’s recommendations, and quality control checks were conducted daily before sample analysis. SPE was performed on the Sebia Hydrasys 2 system using Hydragel 7 Protein (E) (Sebia, France), following the manufacturer’s protocols. Each relative fraction in percentage allowed to calculate concentration on the basis of previous concentration on clinical chemistry analyzer. For most reference data for globulins, fraction identification is inconsistently available. Consequently, the separation of protein into 6 fractions (pre-albumin, albumin, alpha-1 globulins, alpha-2 globulins, beta-globulins, gamma-globulins) was defined in consultation with a board-certified clinical pathologist.

The samples were refrozen up to 3 months after initial tests to preserve them for subsequent analyses, including GM (150 µL), BDG (10 µL), and mannoprotein detection (75 µL).

When sample volume was limited, mannoprotein detection was favored over GM assay. The BDG assay (Fungitell^®^ Assay, Associates of Cape Cod, Inc., East Falmouth, MA, USA) and GM (Platelia^®^ *Aspergillus* EIA, Bio-Rad Laboratories, Marnes-la-Coquette, France) were performed according to the manufacturers’ instructions, with the use of protection of paraffin film above component products to prevent contamination. For GM, due to variability in sample volume, some experiments were performed using adjusted serum-to-diluent ratios, following the manufacturer’s recommendations. To increase the number of analyses while maintaining a consistent ratio between the serum and treatment solution, the following adaptations were made: 70 µL of serum was mixed with 23 µL of treatment solution; when only 125 µL of serum was available, 42 µL of treatment solution was added; and when 100 µL of serum was available, 33 µL of treatment solution was added.

Mannoprotein detection was performed using a lateral flow device (AspLFD test, OLM Diagnostics, Newcaslte, UK), with a recommended serum volume of 150 µL, which was found to be excessive in some cases. An initial trial was performed on six samples (three control, three confirmed) using half the recommended volumes of serum and buffer (75 µL each). This was followed by a standard test using the full recommended volumes, yielding identical results in both cases. As a result, a serum volume of 75 µL was adopted for all subsequent tests. After mixing the serum or plasma with the buffer, the solution was incubated on a heat block at 120 °C for 3 min. Following centrifugation, 70 µL of the supernatant was transferred to the sample port of the test cassette. The device was kept at room temperature, and results were assessed after 30 min. A positive result was indicated by the presence of a precipitation line specific to *Aspergillus*.

### Statistical analysis

#### Description of the study sample

Data entry and consolidation were made using Microsoft Excel 2010^®^ spreadsheet and subsequently analyzed using R (version 4.3.0; R Core Team 2024) [15].

Each bird was characterized by sex, age, respiratory and general clinical signs (lethargy, anorexia/dysorexia, weight loss), 3HB, pre-albumin, albumin, alpha-1 globulins, alpha-2 globulins, beta globulins, gamma globulins concentration, albumin/globulin ratio, GM index and BDG concentration.

First, a descriptive analysis of the sample was performed. Quantitative variables were summarized by classical criteria (mean, standard deviation), and their distributions were explored graphically using histograms and boxplots. Qualitative variables were summarized using contingency tables. The diagnostic performance of the AspLFD test was assessed using contingency tables.

### Performances of diagnostic tests

The unit of analysis was the individual sample. To assess the performance of each diagnostic test under study, which all provided a quantitative response, a receiver operating characteristic (ROC) curve analysis was performed for each test separately, and the corresponding area under the curve (AUC) was calculated. Only samples from birds belonging to groups A (controls) and C (confirmed cases) were here included. Samples from birds in group C were identified on the basis of histological and/or mycological evidence of *Aspergillus* infection, which served as the diagnostic gold standard for defining disease status in the ROC analysis. The Youden index was used to identify an optimal threshold used to binarize the initially quantitative test response: this threshold was determined as the point value where the Youden index (Y = Sensitivity + Specificity − 1) was maximized. In case of repeated sampling, both samples were included.

### Predictive model of aspergillosis state

Principal component analysis (PCA) was performed to explore the distribution of individuals from the dataset used for model and based on selected quantitative variables (3HB, pre-albumin, albumin, alpha-1 globulins, alpha-2 globulins, beta globulins, gamma globulins, and albumin/globulin ratio) to assess whether distinct populations (A, B, and C) could be discriminated. These parameters were selected to maximize cohort size (additional variables such as GM would have reduced the cohort to 80 individuals) and to focus on diagnostic tests readily available in veterinary clinical settings.

Logistic regression models were implemented to predict the likelihood of aspergillosis in birds on the basis of clinical signs and diagnostic tests. The model’s equation is as follows:

$${Y}_{i}\sim Bernoulli\left({p}_{i}\right)$$

$$ln(\frac{{p}_{i}}{1-{p}_{i}})={\upbeta }_{0}+{\upbeta }_{1}{x}_{1i}+\dots$$

where* Y*_*i*_ is the dependent binary variable denoting the presence of aspergillosis in bird *i*, which follows a Bernoulli distribution with proportion *p*_*i*_. *Y*_*i*_ took the value 0 in controls (A) and 1 in cases (C ± B). The log-odds of the probability (also called logit) of aspergillosis was modeled as a linear combination of the different predictors, where *x*_*1i*_ denotes the measured value for predictor *x*_*1*_ in bird *i* and *β*_*1*_ its associated coefficient estimated by the model from the data. The linearity of continuous predictors with the logit of the outcome was assessed graphically. As none of the continuous predictors satisfied this assumption, all were dichotomized according to the thresholds defined in the previous section. The diagnostic tests included in the models as predictor variables were therefore used as binary categorical variables. We initially accounted for the structure effect (e.g., institution or facility) considered as random effect, to control for potential clustering and shared environmental or management factors. This random effect was ultimately found to have no significant influence on the models. Then only fixed-effects regression models were used.

Prior to model fitting, we assessed multicollinearity among predictors using the variance inflation factor (VIF) to ensure stable model coefficient estimates. All included variables had VIF values below 5, indicating low collinearity [16]. A VIF greater than 5 is generally considered indicative of moderate-to-high collinearity with other predictors, and such variables should typically be excluded from the final model to avoid redundancy and instability. The model was optimized through a backward selection process in which the variable with the highest *p*-value was sequentially removed. To account for potential confounding effects, the removal of each variable was evaluated to ensure it did not substantially affect the estimated effects of other variables, with a threshold set at a 20% change. If such a change was observed, the variable was retained in the model, regardless of its statistical significance. The model was then refined through backward selection, removing nonsignificant predictors while retaining variables that acted as confounders. Although the primary goal was prediction, odds ratios (OR) derived from the final logistic regression models were reported to show how each variable was associated with the likelihood of aspergillosis, enhancing interpretability for clinical application.

In a final step, we assessed the overall fit of the final models by computing a marginal *R*^2^ (representing variance explained by the fixed effects). However, in the context where the predicted variable is binary, *R*^2^ can be misleading or not directly applicable. Standard metrics (sensitivity, specificity, AUC) were also computed to evaluate their predictive performance of models [17]. A cutoff value of 0.5 was used to assess discriminatory ability, with predicted probabilities above this threshold considered indicative of aspergillosis.

We hypothesized that suspected cases could be misclassified. Thus, two logistic regression models were developed using the same analytical approach:

- Model 1 compared group A (controls) with a group including both suspected and confirmed aspergillosis cases (groups B + C)
- Model 2 compared group A (controls) with group C (confirmed cases)

This dual approach allowed us to assess whether inclusion of suspected cases influenced the robustness and generalizability of the predictive model.

The variables included in both models encompassed test results (3HB, albumin, alpha-1 globulins, alpha-2 globulins, beta-globulins, gamma-globulins), presence of general clinical signs, presence of respiratory clinical signs, and reported information concerning the animals (age, sex, body condition). Due to insufficient data, mannoprotein detection could not be incorporated into the models, which consequently reduced the total number of eligible individuals. Furthermore, the high variability in BDG measurements and the limited number of birds with available GM results led to the exclusion of these two tests from the analysis. Additionally, both tests are not currently accessible to veterinarians in routine practice. Likewise, not all birds received diagnostic imaging, and thus this variable could not be included in the models. In parallel with the selection of variables, some individuals were excluded if a second sample was taken as part of follow-up for healthy or traumatized birds. Likewise, if two samples of one bird with diagnosis of aspergillosis were available, the sample taken farthest from the final diagnosis of aspergillosis was excluded from the analysis.

On the basis of the comparison of both models, model 2 was chosen for subsequent probability estimation. Predicted probabilities were calculated using the model’s logit equation. As with the determination of thresholds for each test, Youden index was chosen to provide the optimal balance between sensitivity and specificity.

## Results

### Bird population and biological samples

A total of 132 blood samples were collected from 118 aquatic birds from various species, including 101 serum and 31 plasma samples. Among these samples, 92 were obtained from 88 control birds, including 6 birds with traumatic lesions. In total, 17 samples were collected from 7 birds classified as suspected cases, and 23 samples were obtained from 23 birds with confirmed aspergillosis. Details regarding bird species are presented in Table 1. Samples were originating from a veterinary clinic, wildlife rehabilitation center, and four zoological parks in France. Additionally, retrospective samples, up to 4 years old, were requested from these facilities to expand the study sample.

**Table 1:** **Species distribution of the study population (*n* = 118) with mean for each variable separated by species and group**

Species	Groups		3HB			Total protein			Pre-albumin			Albumin			Alpha-1 globulins			Alpha-2 globulins			Total alpha globulins			Beta-globulins			Gamma-globulins			Albumin/globulin ratio			GM			BDG		
			mmol/L			g/L			g/L			g/L			g/L			g/L			g/L			g/L			g/L						Index			pg/mL		
		*n*	Mean	SD	*n*	Mean	SD	*n*	Mean	SD	*n*	Mean	SD	*n*	Mean	SD	*n*	Mean	SD	*n*	Mean	SD	*n*	Mean	SD	*n*	Mean	SD	*n*	Mean	SD	*n*	Mean	SD	*n*	Mean	SD	*n*
Northern gannet (*Morus bassanus*)	A	23	0.47	0.23	22	23.84	12.14	22	0.39	0.21	22	8.7	4.52	22	1.12	0.61	22	7.52	4.48	22	8.64	4.99	22	3.36	2.11	22	2.74	2.18	22	0.68	0.22	22	1.39	2.63	21	198.07	201.17	22
	C	1	0.63		1	11.29		1	0.2		1	4.2		1	0.7		1	2.2		1	2.9		1	3.6		1	0.1		1	0.64		1	10.88		1	713.48		1
Muscovy duck (*Cairina moschata*)	A	2	0.1	0.01	2	45.38	4.73	2	NA		2	23.55	2.19	2	2.1		2	5.45	1.06	2	7.55	1.06	2	9.5	0.57	2	4.95	2.19	2	1.08	0.03	2	0.75	0.13	2	623.64	288.69	2
	C	6	1.78	2.22	5	35.48	2.65	6	0.07	0.16	6	13.03	1.79	6	2.77	0.56	6	7.53	1.48	6	10.3	1.82	6	9.9	2.52	6	2.2	1.06	6	0.61	0.18	6	4.95	0.40	3	777.1	256.72	6
Common guillemot (*Uria aalge*)	A	19	0.49	0.43	19	24.93	19.40	19	0.28	0.10	19	5.82	3.89	19	1.05	1.46	19	10.38	10.46	19	11.44	10.69	19	6.28	7.51	19	1.15	1.73	19	0.51	0.37	19	0.4	0.64	18	255.34	299.12	19
	C	1	1.04		1	43.7		1	0.6		1	4.4		1	0.8		1	18.3		1	19.1		1	15.4		1	4.6		1	0.13		1	0.39		1	161.71		1
Common loon (*Gavia immer*)	A	2	0.42	0.04	2	21.58	0.21	2	0.35	0.07	2	7.9	0.28	2	0.5	0.00	2	6.05	0.78	2	6.55	0.78	2	2.95	0.35	2	3.8	0.85	2	0.62	0.03	2	0.26	0.04	2	36.02	9.94	2
Humboldt penguin (*Spheniscus humboldti*)	A	29	1.05	0.37	27	62.46	11.17	28	0.71	0.27	28	26.22	7.11	28	0.74	0.22	28	10.86	2.20	28	11.6	2.23	28	14.76	6.52	28	8.07	4.74	28	0.81	0.26	28	0.37	0.83	14	230.49	167.04	25
	B	7	0.68	0.26	7	54.52	3.35	7	0.5	0.18	7	17.77	9.67	7	1.16	0.62	7	13.21	2.96	7	14.37	3.39	7	11.14	1.62	7	10.94	2.58	7	0.56	0.36	7	0.25	0.17	4	272.9	149.28	6
	C	14	0.81	0.50	14	57.23	13.74	14	0.67	0.40	13	7.68	3.23	13	1.63	0.61	13	14.63	3.32	13	16.26	3.44	13	15.85	8.63	13	15.76	8.11	13	0.18	0.07	13	0.83	1.09	5	504.92	383.15	10
Razorbill (*Alca torda*)	A	1	2.29		1	48.38		1	0.3		1	9.6		1	0.7		1	16		1	16.7		1	17.1		1	4.2		1	0.26		1	0.46		1	518.23		1
Comb duck (*Sarkidiornis melanotos*)	A	2	0.25	0.06	2	37.4	0.69	2	0.4	0.00	2	17.6	0.28	2	1.95	0.49	2	8.3	0.14	2	10.25	0.35	2	7.8	0.00	2	1.4	0.14	2	0.93	0.01	2	0.3	0.35	2	323.22	184.68	2
Maned duck (*Chenonetta jubata*)	A	1	0.22		1	47.33		1	0.4		1	26.5		1	2.8		1	3.3		1	6.1		1	12.4		1	1.5		1	1.3		1	NA		0	581.24		1
Black-necked swan (*Cygnus melancoryphus*)	A	1	0.11		1	55.04		1	1.4		1	28.5		1	2.9		1	8.7		1	11.6		1	10.2		1	3.2		1	1.19		1	NA		0	452.01		1
Red-crested pochard (*Netta rufina*)	A	6	0.15	0.03	6	47.22	6.98	6	0.52	0.18	6	20.08	5.11	6	2.27	0.60	6	9.63	2.41	6	11.9	2.17	6	9.35	8.93	6	5.15	3.84	6	0.89	0.33	6	0.33	0.25	5	126.99	49.01	6
Pink-backed pelican (*Pelecanus rufescens*)	A	1	0.14		1	12.5		1	0.6		1	5.4		1	0.6		1	2.8		1	3.4		1	1.8		1	0.9		1	0.92		1	0.1		1	26.77		1
European herring gull (*Larus argentatus*)	C	1	0.38		1	44.63		1	0.4		1	9.8		1	1.7		1	20.2		1	21.9		1	9.6		1	3.4		1	0.3		1	NA		0	NA		0
Mandarin duck (*Aix galericulata*)	A	1	0.61		1	37.66		1	0.4		1	18.1		1	1.8		1	8.5		1	10.3		1	7.8		1	1.4		1	0.97		1	NA		0	NA		0

Animals were classified as group A (control), group B (suspect), and group C (confirmed).

3HB: 3-hydroxybutyrate, GM: galactomannan, BDG: beta-d-glucan

Although a total of 118 birds were initially enrolled, not all diagnostic tests were performed on every individual bird, leading to sample size fluctuations when assessing the test performances.

### Performances of diagnostic tests

#### Galactomannan and beta-D-glucan

Only 80 samples provided sufficient serum or plasma volume for GM analysis. The resulting AUC was 0.72, with a sensitivity of 55%, specificity of 86%, and a Youden index of 0.40 with a threshold of 0.73 index (Table 2).

**Table 2:** **Performance of each blood test with detailed sample size**

Analysis	Unit	*N* =	AUC	Se	Sp	TPP	Thresholds	Youden index
ß-d-glucan	pg/mL	107	0.80	53%	94%	10/19	624.89	0.47
Galactomannan	Index	80	0.72	55%	86%	6/11	0.73	0.4
Gamma-globulins	g/L	114	0.75	61%	89%	14/23	7.95	0.50
Albumin/globulin ratio	No unit	114	0.86	83%	75%	19/23	0.48	0.57
Beta globulins	g/L	114	0.73	96%	51%	22/23	6.90	0.46
Total alpha globulins	g/L	114	0.69	52%	84%	12/23	14.85	0.36
Alpha-2 globulins	g/L	114	0.65	48%	87%	11/23	14.05	0.35
Alpha-1 globulins	g/L	114	0.78	83%	64%	19/23	0.95	0.46
Total albumin	g/L	114	0.65	100%	46%	23/23	14.90	0.46
Albumin	g/L	114	0.65	100%	45%	23/23	14.85	0.45
Pre-albumin	g/L	114	0.50	48%	60%	11/23	0.45	0.08
3-hydroxybutyrate	mmol/L	114	0.63	78%	52%	18/23	0.52	0.29

AUC: area under the curve, Se: sensitivity, Sp: specificity, TPP: prediction of positive individuals are predicted birds among confirmed group (C).

For BDG, the serum quantity was sufficient for 107 samples. AUC was 0.80, with a sensitivity of 53% and a specificity of 94%, resulting in a Youden index of 0.47 with a threshold of 624.89 pg/mL (Table 2). Two samples could not be analyzed due to hemolysis.

#### Mannoprotein detection

The test was performed on 79 blood samples. Two versions of the test interpretation were evaluated. Because line 1 was occasionally faint or barely visible, an alternative scoring system was implemented to enhance result interpretation. On the basis of the visibility of the precipitation line, the initial scoring system yielded a sensitivity of 50% and a specificity of 85% when a score of 0 was considered negative and scores of 1, 2, and 3 were considered positive. When both scores 0 and 1 were classified as negative, sensitivity decreased to 40%, while specificity increased to 100%.

#### 3-hydroxybutyrate

The test was performed on 114 blood samples. AUC was 0.63, with a sensitivity of 78% and a specificity of 52%, resulting in a Youden index of 0.29 with a threshold of 0.52 mmol/L (Table 1).

#### Protein electrophoresis

**Figure 1:**
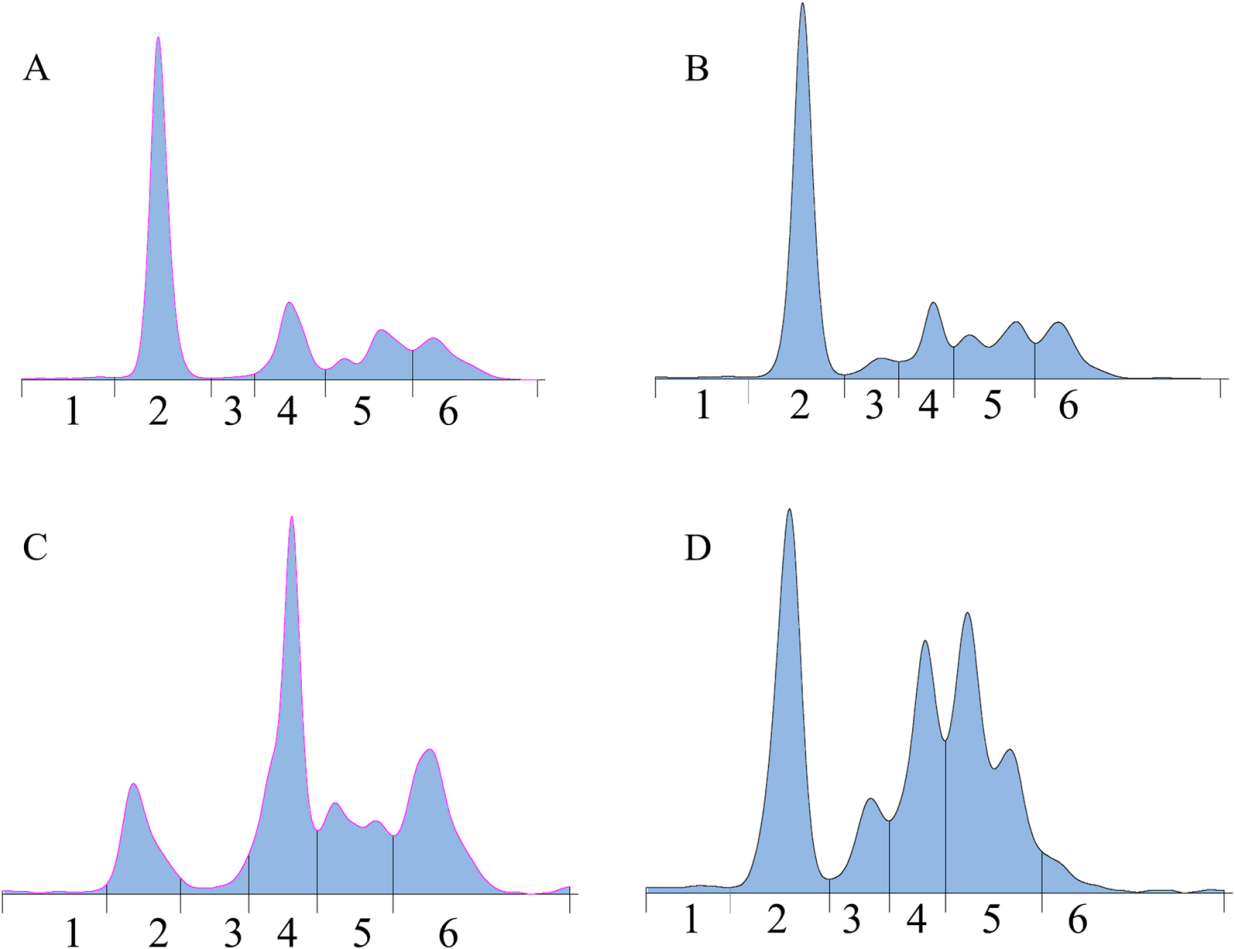
**Electrophoresis profile of fraction protein separation of clinically healthy (A and B) and confirmed infected by *Aspergillus* (C and D) Humboldt penguins (*Spheniscus humboldti*) and Muscovy ducks (*Cairina moschata***), respectively. 1: pre-albumin, 2: albumin, 3: alpha-1 globulins, 4: alpha-2 globulins, 5: beta-globulins, 6: gamma-globulins

Serum protein electrophoresis was performed on plasma (*N* = 29) or serum (*N* = 85) samples from 114 birds. The Youden index ranged from 0.08 (pre-albumin) to 0.57 (albumin/globulin ratio). The best performance was observed for the albumin/globulin ratio, with AUC of 0.86, sensitivity of 83%, and specificity of 75%, with a threshold of 0.48. The detailed results are presented in Table 2. Example of protein separation is available in Figure 1.

### Predictive model of aspergillosis state

A total of 112 birds were included in the logistic regression analysis, as only individuals with complete data were eligible.

**Figure 2:**
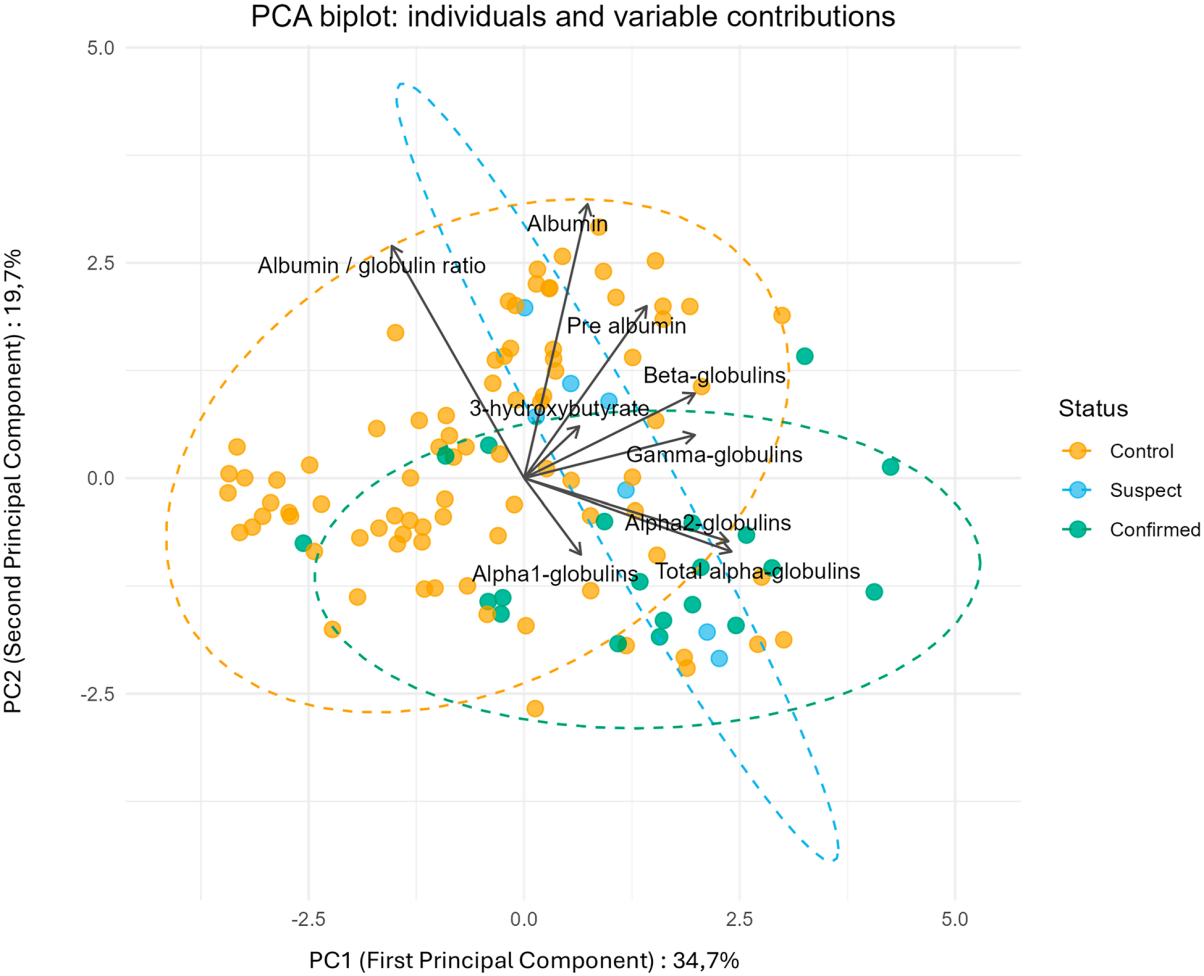
**Principal component analysis (PCA) of 112 birds included in logistic regression analyses.** Group A (control) is represented in orange, group B (suspect) in blue, and group C (confirmed) in green, highlighting the distinct clusters observed in the data. Selected quantitative variables were the following: 3HB, pre-albumin, albumin, alpha-1 globulins, alpha-2 globulins, beta globulins, gamma globulins, and albumin/globulin ratio. Arrows indicate the contribution (loading) of each variable to the principal components.

The PCA results (Figure 2) revealed that suspected cases were positioned between confirmed cases and healthy individuals, suggesting an intermediate clinical and biochemical profile. Suspected cases were mixed between control and confirmed cases in agreement with the hypothesis of a misclassification of these cases. Therefore, the two separate models were well developed to refine the analysis.

Univariable analyses were performed using type III analysis of variance (ANOVA) on individual predictors. Each predictor variable was coded as binary categorical variables using thresholds defined above for inclusion in both model 1 and model 2. As the population differed between the two models, analyses were conducted separately for the population of model 1 and that of model 2, which allowed for the removal of pre-albumin from both models. The results are provided in Additional file 1.

Model 1 compared control birds (*n* = 85) and birds with aspergillosis. Suspected cases (*n* = 7) were associated with confirmed cases (*n* = 20). This model identified several significant predictors of aspergillosis, including body condition score, presence of respiratory clinical signs, 3HB, beta-globulins, and gamma-globulins concentrations. Gamma-globulins concentration above 7.95 g/L was strongly associated with the occurrence of aspergillosis (OR 39.7, 95% CI 4.55–1231.83, *p* = 0.006). Similarly, 3HB concentration above 0.52 mmol/L was significantly associated with confirmed and suspected aspergillosis (OR 22.8, 95% CI 2.42–714.98, *p* = 0.022). Beta-globulin levels exceeding 6.90 g/L were also associated with elevated odds of disease, but were not significant (OR 18.3, 95% CI 1.13–1061.41, *p* = 0.078). The presence of respiratory clinical signs was a strong predictor, with an OR of 85.8 (95% CI 9.66–2353.83, *p* < 0.001), while a decreased body condition score was similarly linked to a higher likelihood of aspergillosis (OR 38.9, 95% CI 4.41–1184.38, *p* = 0.006). These results are summarized in Table 3.

**Table 3:** **Predictor variables associated with aspergillosis state and corresponding odds ratio of the multivariate logistic fixed-effects regression model 1**

Variable	Coefficient (log-odds)	95% CI (log-odds)	Odds ratio	95% CI (OR)	*p*-Value
Intercept	−10.44	[−19.07 to −5.96]	2.90^E^–05	[0–0]	< 0.001
Body condition < 3/5	3.66	[1.48–7.08]	38.94	[4.41–1184.38]	0.006
Presence of respiratory clinical signs	4.45	[2.27–7.76]	85.78	[9.66–2353.83]	< 0.001
3-hydroxybutyrate > 0.52 mmol/L	3.13	[0.89–6.57]	22.83	[2.42–714.98]	0.022
Beta-globulins > 6.90 g/L	2.91	[0.12–6.97]	18.34	[1.13–1061.41]	0.078
Gamma-globulins > 7.95 g/L	3.68	[1.51–7.12]	39.7	[4.55–1231.83]	0.006

Model 2 compared control birds without aspergillosis (*n* = 85) with birds with confirmed aspergillosis (*n* = 20). Birds with suspected aspergillosis (*n* = 7) were excluded. In model 2, 3HB, alpha-1 globulins, beta globulins concentrations, and both respiratory and general clinical signs were retained as significant predictor variables. Model 2 showed that birds with beta-globulin concentrations exceeding 6.90 g/L had significantly higher odds (OR 135.52, 95% CI 6.54–12,908.5, *p* = 0.00875) of developing aspergillosis (Table 4). Elevated 3HB concentrations (> 0.52 mmol/L) (OR 38.43, 95% CI 2.68–2445.27, *p* = 0.02826) and the presence of respiratory clinical signs (OR 34.41, 95% CI 2.75–1193.43, *p* = 0.01602) were also identified as significant predictor variables associated with an increased likelihood of aspergillosis. In addition, the presence of general clinical signs (OR 66.81, 95% CI 5.64–2537.67, *p* = 0.00419) and alpha-1 globulin levels greater than 0.95 g/L (OR 24.65, 95% CI 2.54–627.38, *p* = 0.01431) emerged as significant predictor variables for aspergillosis in this model (Table 4).

**Table 4:** **Predictor variables associated with aspergillosis state and corresponding odds ratio of the multivariate logistic fixed-effects regression model 2**

Variable	Coefficient (log-odds)	95% CI (log-odds)	Odds ratio	95% CI (OR)	*p*-Value
Intercept	− 12.35	[−21.94 to −7.01]	4.00^ × 10^–6	[0–0]	< 0.001
Presence of respiratory clinical signs	3.54	[1.01–7.08]	34.41	[2.75–1193.43]	0.01602
Presence of general clinical signs	4.2	[1.73–7.84]	66.81	[5.64–2537.67]	0.00419
3-hydroxybutyrate > 0.52 mmol/L	3.65	[0.99–7.8]	38.43	[2.68–2445.27]	0.02826
Alpha-1 globulins > 0.95 g/L	3.2	[0.93–6.44]	24.65	[2.54–627.38]	0.01431
Beta globulins > 6.90 g/L	4.91	[1.88–9.47]	135.52	[6.54–12,908.5]	0.00875

The estimated coefficients presented in Table 4 can be used to predict the probability of aspergillosis in individual birds using the following formula, derived from the logistic regression model equation:

$${\mathrm{p}}_{\mathrm{i}}=\frac{exp\left({\upbeta }_{0}+{\upbeta }_{1}{x}_{1i}+... \right)}{1+exp\left({\upbeta }_{0}+{\upbeta }_{1}{x}_{1i}+...\right)}$$

The final logistic regression equation for Model 2 is presented below.

$${\mathrm{p}}_{\mathrm{i}}=\frac{exp\left(-12.35+3.54\cdot SCresp,i+4.20\cdot SCgen,i+3.65\cdot HBi+3.20\cdot \alpha 1i+4.91\cdot \beta i \right)}{1+exp\left(-12.35+3.54\cdot SCresp,i+4.20\cdot SCgen,i+3.65\cdot HBi+3.20\cdot \alpha 1i4.91\cdot \beta i\right)}$$

This formula provides a probability between 0 and 1 that the aquatic bird is affected by aspergillosis, based on its clinical signs and biochemical alterations. For a given bird ($i$), replace $SCresp,i$ with 0 (absent) or 1 (present) to indicate the presence of respiratory clinical signs, $SCgen,i$, with 0 or 1 for the presence of general clinical signs, *HB*_*i,*_ (β-hydroxybutyrate > 0.52 mmol/L) with 0 or 1 depending on whether the threshold is exceeded and and similarly for *α *_*i,*_ (α₁-globulins > 0.95 g/L), *ß*_*i*_ (β-globulins > 6.90 g/L). Using the optimal Youden index to define a classification threshold, birds with predicted probabilities above 0.30 were considered “probable aspergillosis,” while those below were considered “unlikely.” This threshold yielded a sensitivity of 95% and a specificity of 95% for identifying aspergillosis in this population.

To compare model performance, both models were applied to populations A and C. Both models exhibited strong discriminatory performance for predicting aspergillosis. Model 1 achieved AUC of 0.98, with sensitivity of 98% and specificity of 80%. Prediction of individuals were accurate for 83/85 individuals of group A and 16/20 animals for group C.

In comparison, Model 2 showed a slightly lower AUC of 0.97 when applied without suspected birds (*n* = 105), but achieved a higher sensitivity (99%) and similar specificity (80%). The *R*^2^ for model 2 was 0.76. With this model, applied on group B, four of them were attributed as sick of aspergillosis, and three as healthy. Prediction of individuals for model 2 were accurate for 84/85 individuals in group A and 16/20 animals in group C.

Overall, models 1 and 2 demonstrated a similar overall fit, as reflected by their performance, although model 1 provided slightly better discrimination (AUC and specificity). In addition, model 2 demonstrated a slightly higher accuracy and *R*^2^ compared with model 1 (Table 5).

**Table 5:** **Comparison of model performance with and without suspects**

Model	AUC	Se	Sp	*R*^2^	Accuracy
Model 1 on population A and C (*n* = 105)	0.98	0.98	0.80	0.71	0.94
Model 2 on population A and C (*n* = 105)	0.97	0.99	0.80	0.76	0.95

## Discussion

Our objectives were to evaluate the accuracy of different blood tests in detecting aspergillosis in birds, to develop logistic regression models to support diagnostic decision-making, and to determine whether selected subsets of clinical and laboratory variables could be used as predictors of disease state. These models were developed following the evaluation of each diagnostic test, using thresholds determined in those analyses. Our findings demonstrate that it is possible to estimate the probability of aspergillosis using selected predictive variables. In model 1, key predictors included body condition score, the presence of respiratory clinical signs, 3HB, beta-globulins, and gamma-globulins. Model 2 incorporated 3HB, alpha-1 globulins, beta-globulins, and both respiratory and general clinical signs.

### Population recruitment

Our study population showed substantial interspecies variability, with representatives from several avian zoological groups. Sphenisciformes were the most represented order, although only one species was included (*Spheniscus humboldti*). The other species included belonged to the following orders: Anseriformes (*Aix galericulata, Cairina moschata*, *Chenonetta jubata, Cygnus melancoryphus*, *Netta rufina*, *Sarkidiornis melanotos*), Charadriiformes (*Alca torda*, *Larus argentatus, Uria aalge*), Suliformes (*Morus bassanus*), Gaviiformes (*Gavia immer*), and Pelecaniformes (*Pelecanus rufescens*). Although these species can be grouped as aquatic birds, they display various characteristics in their habitats, or molt status, which could be reflected in their biological values [18, 19]. This could impact test results, particularly electrophoresis, as total protein concentrations can differ among species [20, 21]. In addition, the various facilities showed differences in data collection methods. Specifically, the age, and in some cases, the sex of birds at the wildlife center could not be confirmed. As a result, age was primarily estimated on the basis of species-specific characteristics, such as plumage and beak appearance. In addition, obtaining only serum was not always feasible depending on the facility, which could influence the data, as serum and plasma composition varies [22]. Some samples were also hemolyzed, which may affect electrophoresis or BDG analysis [23, 24]. Although repetitive freeze–thaw cycles could potentially affect assay performance, previous studies have demonstrated the stability of galactomannan in frozen samples. A retrospective evaluation of the Platelia GM test showed consistent results after 5 years of storage at −20 °C [25]. However, the manufacturer recommends limiting freeze–thaw cycles to a maximum of four to maintain optimal test accuracy. In our study, samples underwent three freeze–thaw cycles, which remains within acceptable limits and is unlikely to have significantly impacted our results. However, thawing may have had an impact on BDG results, as the manufacturer recommends storing samples frozen at −20 °C for up to 27 days or at −80 °C for up to 4 years. The effect of multiple freeze–thaw cycles on Asp-LFD performance has not been evaluated. In contrast, extended freezing may have an impact on protein concentration [26, 27]. A limitation of the present study is the unequal representation of species among groups. Several species were represented exclusively in group A, without corresponding individuals in groups B or C. This imbalance restricted intergroup comparisons for those taxa. Because baseline values for protein fractions and metabolite concentrations vary across avian species, the inclusion of taxa represented only in group A may have increased within-group variability and reduced between-group discrimination. However, excluding these individuals would have substantially decreased the overall sample size (*n* = 112) and statistical power, resulting in less stable and less robust model estimates. All individuals were therefore retained to preserve model stability.

### Test performance

GM and BDG were included in the present study as they mark the development of *Aspergillus* fungi. Previous studies on GM detection using the Platelia^®^ test (Bio-Rad Laboratories) have shown variable sensitivity, ranging from 7% to 86% in birds with aspergillosis, with cutoff values between 0.5 and 2 [28]. Our study found a cutoff of 0.73, resulting in a sensitivity of 55%, which remains relatively low. Similarly, BDG was tested in a single larger cohort of birds consisting of various species, showing sensitivity of 60% and specificity of 93%, with a threshold of 461 pg/mL. This threshold was lower than the 624.89 pg/mL observed in our cohort, where sensitivity was 55% [29]. These markers do not yield consistent results across studies and exhibit considerable variability. Moreover, these markers require specialized equipment that is neither available in veterinary clinics nor in most specialized veterinary laboratories, making them nonrelevant for routine use. In addition, the analysis process is time consuming and labor intensive, further limiting their utility. The growing availability of point-of-care tests enabling targeted, on-demand measurements could improve the routine use of such markers.

An interesting test for detecting *Aspergillus* fungi is mannoprotein detection via immunochromatography using the *Aspergillus* lateral-flow device (AspLFD, OLM Diagnostics, Braintree, UK). The Asp-LFD can detect mannoprotein in 30 min, and appears promising for routine use with low blood volume. A previous study reported a sensitivity ranging from 4% to 76% in a population of 23 captive Gentoo penguins [12]. In the present study, sensitivity ranged from 40% to 50% in a larger cohort, with improved specificity (85–100%). However, interpreting positive results was challenging when the precipitation line appeared faint. To address this, we conducted a second analysis, considering advanced stages of the test (levels 2 and 3) as positive, which allowed for better interpretation of the results.

Protein electrophoresis and 3HB were selected as accessible markers for veterinarians due to their nonspecific yet practical diagnostic potential. In the present study, 3HB was evaluated as a nonspecific marker for aspergillosis, with a threshold set at 0.35 mmol/L, yielding a sensitivity of 95% and specificity of 33%. In comparison, a previous study in birds reported average test performance (sensitivity = 65%, specificity = 79%) with a threshold of 0.94 mmol/L [30]. It is important to note that this biomarker is not specific to aspergillosis and may be elevated in cases of weight loss and anorexia. Body condition scores were requested from the medical records of various facilities to account for these factors; however, incomplete or unclear documentation precluded a thorough analysis of their impact on 3HB.

The albumin/globulin ratio demonstrated notable diagnostic performance in our study population, with an estimated sensitivity of 83% and specificity of 75% with an AUC of 0.86, which is the best performance of a test used alone in the present study. Our optimal threshold of 0.48 differed from the previously reported value of 0.96, which was associated with a sensitivity of 70% and a specificity of 75% [31]. Although the analytical devices differed between the two studies, both used agarose gel electrophoresis rather than capillary systems. In addition, the previous study analyzed plasma only, whereas our study combined plasma and serum samples. Previous research has shown that, in birds, electrophoretic results for the same sample can vary depending on whether capillary or gel electrophoresis is used [32, 33]. It is important to note that concentration levels and gel separation techniques can vary across species. Moreover, alpha-1 globulins were defined arbitrarily, using alpha-2 globulins as reference, and electrophoresis profiles are not available in literature for all bird species. Therefore, threshold concentrations should ideally be established for each species separately, rather than using a unique threshold common for all species.

### Prediction of aspergillosis state

Logistic regression models have proven useful to predict disease in animals and human medicine [34, 35] and have also been applied to retrieve risk factors associated to avian influenza in wild birds [36], supporting their relevance for improving disease surveillance. Similar applications remain scarce for disease prediction in veterinary medicine, and even more in avian medicine. This gap highlights the potential value of exploring statistical tools such as logistic regression to improve diagnostic approaches for diseases in wildlife and captive populations.

Both models identified the presence of respiratory clinical signs, elevated 3HB levels, and increased globulin fractions as key predictors. Specifically, 3HB and respiratory clinical signs consistently emerged as significant variables across both models. These findings are in line with previously published data, although the thresholds reported in earlier studies differ from those identified here [13, 28, 30]. The association between respiratory signs and aspergillosis is expected, as the respiratory tract is typically the primary site of colonization and disease development following inhalation of *Aspergillus* spores. Although confidence intervals were wide for both models, likely due to the small number of individuals or uneven group sizes, the odds ratios remained high, suggesting strong and biologically meaningful associations.

Protein electrophoresis has proven to be a valuable tool for both the diagnosis and monitoring of aspergillosis and is commonly employed in avian clinical practice [10, 28, 37, 38]. In contrast to earlier studies, however, the albumin-to-globulin (A/G) ratio was not retained in our final predictive models. This discrepancy may be attributed to its collinearity with beta-globulin levels, which could reduce its independent predictive value.

It is noteworthy that when used individually, both beta-globulins and 3HB demonstrated a specificity of only 51%, highlighting the importance of multivariate approaches for improving diagnostic accuracy. Finally, although one model included alpha-1 globulins and the other gamma-globulins, only seven individuals differed between the two datasets, preventing any firm conclusions regarding the discriminative value of these specific protein fractions. Overall, the performance of the two models was comparable, suggesting that the core set of predictive variables remains robust across slight variations in dataset composition. Confidence intervals for odds ratios in all variables were wide for both models. A wide 95% confidence interval indicates low precision in the estimated odds ratios; nevertheless, the relatively high ORs suggest that the associations are biologically plausible. To enhance clinical applicability, the variables retained in model 2 can be combined to estimate the probability that an individual bird has aspergillosis, according to the logistic regression equation. On the basis of the model coefficients, birds exhibiting both respiratory and general clinical signs together with biochemical alterations (3 HB > 0.52 mmol/L, alpha-1 globulins > 0.95 g/L, beta-globulins > 6.9 g/L) have a markedly increased predicted probability of aspergillosis, whereas birds lacking these abnormalities have a very low estimated probability. This model demonstrated good predictive performance, correctly classifying most birds, with high sensitivity and specificity, indicating its potential utility for identifying aspergillosis in this population. Ideally, the predictive performance of this model should be validated on an independent population of birds to confirm its robustness and generalizability.

However, these findings should be interpreted with caution. The limited sample size and unbalanced group distribution contributed to the uncertainty around this estimate, as reflected by the wide confidence intervals. Additional validation using an independent dataset is necessary to confirm the model performance. Future studies should ideally include a more balanced species distribution or perform species-specific analyses once sufficient sample sizes are available. It should be noted that diagnostic imaging tests, which are commonly used in clinical practice and could contribute to optimal diagnosis, were not included in the predictive models. Although such data were requested from the veterinarians, their inclusion was not possible because imaging was not performed for all animals in the cohort, even though some lesions were observable in a few cases.

In summary, the findings of this study indicate that clinically infected aquatic birds exhibit elevated levels of beta-globulins and 3HB compared with healthy individuals. Given the nonspecific clinical presentation of aspergillosis, it is crucial to improve diagnostic accuracy through accessible and reliable clinical tests. Further investigations are needed to evaluate the specificity of these biomarkers by comparing birds affected by other infectious diseases. In addition, novel biomarkers may be identified through omics-based approaches, potentially enabling the development of more sensitive assays to confidently detect subclinical infections at early stages of the disease.

## Supplementary Information

**Additional file 1** **Univariable analyses were performed using type III ANOVA on individual predictors (age, sex, body condition score, hydroxybutyrate, pre-albumin, albumin, alpha-1 globulins, alpha-2 globulins, beta globulins, gamma globulins, albumin/globulin ratio).**

## Data Availability

The data presented in this study are available upon request from the corresponding author.
