# Supplementary material for: Assessment of the performances of blood tests for the antemortem diagnosis of aspergillosis in wild or captive aquatic birds
Source: Vet Res. 2026 Feb 3;57:38. doi: 10.1186/s13567-026-01711-3 (PMC13130812; doi:10.1186/s13567-026-01711-3)
Supplement: Supplementary file 1 — Additional file 1 Univariable analyses were performed using type III ANOVA on individual predictors (age, sex, body condition score, hydroxybutyrate, pre-albumin, albumin, alpha-1 globulins, alpha-2 globulins, beta globulins, gamma globulins, albumin/globulin ratio). [file 13567_2026_1711_MOESM1_ESM.docx]

# Additional file 1: Univariable analyses

**Control (A) vs confirmed (C)**

| Variable | LR Chisq | P_value |
| --- | --- | --- |
| Age | 6.8149 | 0.00904 |
| Sex | 8.8014 | 0.01227 |
| Body Condition Score | 14.352 | 0.0001516 |
| Hydroxybutyrate | 8.0487 | 0.004554 |
| Pre-albumin | 0.6585 | 0.4171 |
| Albumin | 21.282 | 3.965e-06 |
| Alpha-1 globulins | 14.863 | 0.0001156 |
| Alpha-2 globulins | 15.451 | 8.468e-05 |
| Beta globulins | 17.032 | 3.675e-05 |
| Gamma globulins | 22.923 | 1.686e-06 |
| Albumin/globulin ratio | 26.611 | 2.488e-07 |

**Control (A) VS suspect (B) and confirmed (C)**

| Variable | LR Chisq | P_value |
| --- | --- | --- |
| Age | 4.7351 | 0.02955 |
| Sex | 13.858 | 0.000978 |
| Body Condition Score | 6.9492 | 0.008386 |
| Hydroxybutyrate | 8.831 | 0.002962 |
| Pre-albumin | 1.1665 | 0.2801 |
| Albumin | 6.9492 | 0.008386 |
| Alpha-1 globulins | 12.966 | 0.0003172 |
| Alpha-2 globulins | 13.498 | 0.0002389 |
| Beta globulins | 23.705 | 1.123e-06 |
| Gamma globulins | 33.722 | 6.358e-09 |
| Albumin/globulin ratio | 22.339 | 2.285e-06 |
